# Digitalization?A Matter of Trust: A Double-Mediation Model Investigating Employee Trust in Management Regarding Digitalization

**DOI:** 10.1007/s11846-022-00598-6

**Published:** 2022-11-23

**Authors:** Angelika Lau, Mona Höyng

**Affiliations:** Department of Human Resource Management, University of Duisburg-Essen, Mercator School of Management, Lotharstraße 65, LB 013c, 47057 Duisburg, Germany

**Keywords:** Digitalization, LMX, Organizational politics, Trust in management, Vision

## Abstract

The purpose of this paper is to determine how employee trust in management regarding digitalization (TMD) is encouraged to successfully promote technological change linked to digitalization and implement digital technologies within organizations. TMD is considered a necessary precondition for employee cooperation regarding the successful implementation of digitalization within organizations. Derived from existing theoretical and empirical research on trust, a double-mediation model is developed. The proposed model investigates the direct relationships between strong digital vision (SDV), leader-member exchange (LMX), and perceptions of organizational politics (POP) on employee TMD. Further, the mediating roles of LMX and POP are investigated regarding the relationship between SDV and TMD. Based on data collected from 1,145 employees of an internationally operating energy supplier, significant positive relationships between SDV, LMX, POP, and employee TMD were found. Further, the results confirmed that LMX and POP sequentially double-mediated the relationship between SDV and TMD. Consequently, by developing a theoretical model for the specific context of digitalization, this study contributes to theory development concerning employee TMD. Furthermore, this study provides practical implications for management in terms of identifying institutional aspects within organizations that enhance TMD in the digital labor context.

## Introduction

In the digital era, organizations increasingly rely on their employees to successfully promote technological change linked to digitalization (Schneider and Sting 2020). Thereby, digitalization is the adaption and use of digital technologies in an organizational context (Legner et al. 2017; Tilson et al. 2010). The use of digital technologies reshapes work practices, to a large and unpredictable extent, and enables new forms of work, such as working from home, which became particularly significant during the COVID-19 pandemic (Bartsch et al. 2020; Timonen and Vuori 2018).

Nevertheless, because digitalization can lead to new forms of indirect and algorithmic control, it might stoke employees’ fears that this technology is threatening their autonomy or can be abused for enabling exploitative and unequal working conditions (Mengay 2020). Therefore, digitalization is associated with an unleashed crisis of trust, as traditional institutional and interpersonal logistics are not attuned to dealing with the risks linked to the prevalence of digital technologies (Bodó 2021).

In this regard, the degree of trust is a critical factor influencing the way employees feel, think, and behave with regard to a particular technological change and is a key component regarding employees’ technology acceptance and adaption (Bahmanziari et al. 2016; Smollan 2013). Especially in the context of digitalization, employee trust in management who lead digitalization is considered a necessary precondition for employees’ cooperation and success regarding the implementation of digitalization (Kotter 1995; Shah et al. 2017; van Dam et al. 2008). As employees must continuously adapt to these changes to keep up with the changing work environment (Shah et al. 2017; Ulrich and Yeung 2019), employee trust in management is a key factor to achieve desirable individual and workplace outcomes (Yunus and Mostafa 2021), such as reducing employees’ resistance to change (Vakola 2014).

Hence, to provide practical implications for meeting this corporate challenge (e.g., Vakola 2014), employee trust in management regarding digitalization (TMD) is of particular interest in the digital labor context. In line with past research, TMD in our study is described as a macro-level view that is a driving force for organizational change (Fulmer and Gelfand 2012; Gibson and Birkinshaw 2004).

Although there has been a growing interest in trust in management (e.g. Fulmer and Gelfand 2012; Schoorman et al. 2007), little is known about the factors that increase employee TMD. In particular, research focusing on the dynamics of trusting relationships between individuals in an organization affected by technological interventions, such as digitalization, is greatly needed (Schafheitle et al. 2020). While research in the field of trust in technology, organizational trust, and technology acceptance is constantly expanding (Bodó 2021; Meeßen et al. 2019), a theoretical framework that investigates the antecedents of employee TMD and their interrelations is lacking.

To address these research gaps, we derive a theoretical framework to develop a double-mediation model that investigates the antecedents of employee TMD in a digital labor context. Therefore, we focus on institutional aspects that are under-explored in the context of trust (Bodó 2021) but are derived as necessary preconditions for TMD.

The current study makes the following contributions to existing literature. To our knowledge, this is the first empirical analysis of employee TMD by considering and applying prior research to the specific digital labor context. In particular, we consider TMD to be affected by institutional aspects, such as roles, rules, and structures in the organization (Fox 1974; McCauley and Kuhnert 1992) in terms of strong digital vision (SDV), leader-member exchange (LMX), and perceptions of organizational politics (POP). By doing so, our study contributes to theory development concerning TMD and provides crucial managerial recommendations in terms of identifying institutional aspects that are highly relevant within organizations to enhance employee TMD in the digital era.

## Theoretical background and hypotheses development

As regards the digital labor context focused on in our study, the concept of TMD is defined based on research by Fulmer and Gelfand (2012), Mayer et al. (1995), and Stanley et al. (2005), among others. There is no universal conceptualization of, or method of measuring trust in management (Mayer et al. 1995; Stanley et al. 2005). However, Mayer et al. (1995) found that willingness to assume risk is common to most conceptualizations. Thus, we define TMD not as taking risk per se, because it is more a willingness to do so (Mayer et al. 1995; Stanley et al. 2005). Moreover, following prior research (Driscoll 1978; Mayer et al. 1995), TMD refers to trust at the individual level, whereby the employee (trustor) trusts in their organizational management (trustee) regarding digitalization (Fulmer and Gelfand 2012). More precisely, employees who trust the management regarding digitalization are willing to make themselves vulnerable to potential negative consequences resulting from the management’s decisions or actions in this regard (Mayer et al. 1995).

As elucidated above, derived from Bodó (2021) and McCauley and Kuhnert (1992), among others, we address existing research gaps and identify institutional aspects as antecedents of TMD. We argue that such aspects are of particular importance in the digital labor context, since employees perceive that organization-wide characteristics, such as digitalization processes, are controlled by management (Bodó 2021; McCauley and Kuhnert 1992). In doing so, we build on the model of trust (Mayer et al. 1995) as theoretical framework, investiagte crucial factors of trust in the digital labor context and integrate existing theoretical and empirical research to derive our research model.

Following the model of trust and research by Colquitt et al. (2007), we argue that employee TMD is determined by the management’s *ability, benevolence*, and *integrity*. Applying these dimensions to the context of the current study, ability is investigated as the perception that management has the skills and competencies to communicate a collective vision of digitalization in terms of SDV (Davis et al. 2000; Fulmer and Gelfand 2012; McCauley and Kuhnert 1992). Hence, SDV is an important external condition in terms of institutional structure which produces common knowledge and shared expectations regarding digitalization and is an important factor in the development of trust in management (Bodó 2021). Thus, SDV is assumed to increase TMD.

Next, we examine benevolence as the employee’s perception that the leader provides support, loyalty, and openness regarding their feelings of belonging (Fulmer and Gelfand 2012). We assume that benevolent leaders maintain high-quality LMX, which promotes TMD (Colquitt et al. 2007; Fulmer and Gelfand 2012; Mayer et al. 1995; McCauley and Kuhnert 1992). While the management’s personal relation to employees is seen as limited, the employee is in contact with their leader. This particular relationship, in terms of LMX, is considered an important institutional aspect in the context of the management’s responsibility and is thus highly relevant for TMD (Fox 1974; McCauley and Kuhnert 1992).

Lastly, management’s integrity is investigated by POP in our model as it refers to the fairness and justice principles within an organization which are deemed acceptable by the employee (Boudrias et al. 2021; Colquitt et al. 2007; Fulmer and Gelfand 2012; McCauley and Kuhnert 1992). This means that even when an employee does not share the management’s viewpoint on digitalization, they might still trust management when they believe it is honest and fair (Davis et al. 2000). Thus, POP is an important organization-wide variable in the development of trust in management (McCauley and Kuhnert 1992) and considered in our model.

Besides, while the model of Mayer et al. (1995) provides a fundamental theoretical framework for our model, we emphasize the need for a new and complex model when it comes to the development of TMD and have consequently created a double-mediation model. Therefore, we extend the existing model of trust by arguing that the selected variables are in a specific relationship with each other instead of being independent antecedents of TMD. This extension addresses a research gap regarding TMD and combines findings of different research areas, which are described in more detail below.

### SDV and TMD

Derived from prior research, we argue that the articulation of an SDV fosters employee TMD. Thereby, an SDV affects an employee’s perceptions that fair formal procedures take place within the digital transformation process of the organization, and provides a direction for employees (Niehoff and Moorman 1996). Following Choi (2007), the perception of an SDV is a key to successfully implement change related to digitalization within organizations (Choi 2007). Thereby, an SDV perceived by employees is associated with a changing future linked to the adaption and use of digital technologies in the organizational context (Hagberg et al. 2016; Legner et al. 2017; Matt et al. 2015; Tilson et al. 2010). Hence, SDV has the power to develop a sense of collective identity among employees, and fosters their trust in management (Den Hartog et al. 2002). This is in line with Uhl-Bien et al. (2000) who state that high levels of trust result in the internalization of shared values. Consequently, we hypothesize:

*H1: Strong digital vision is positively related to trust in management regarding digitalization.*

### The mediating role of LMX

Grounded on social exchange theory (Blau 1964), LMX-theory focuses on the dyadic and unique relationship between a leader and an employee that develops over time (Bauer and Erdogan 2016). Specifically, LMX is described as an exchange-based relationship between a leader and a follower, whereby the quality of this relationship determines outcomes at the individual, group, and organizational level (Gerstner and Day 1997). High-quality LMX relationships are of particular importance to this study as they are associated with outstanding interpersonal relationships, mutual influence, and the internalization of common goals (Breevaart et al. 2015; Deluga and Perry 1991; Graen and Uhl-Bien 1991, 1995).

Referring to the digital labor context, we argue that an SDV promotes high-quality LMX. In particular, we argue that benevolent leaders maintain high-quality dyadic relationships and attachments between themselves and their employees (Colquitt et al. 2007; Mayer et al. 1995; Nazir et al. 2021). In this regard, Kauppila (2016) found that sharing a vision fostered interpersonal relationships and reduced employees’ anxiety by offering information and creating a sense of community (Kim et al. 2005). Furthermore, sharing a digital vision fosters the development of a sense of collective identity. Thereby, identification-based trust increases (Den Hartog et al. 2002), and interpersonal relationships are enhanced (Kauppila 2016) in terms of high-quality LMX. Thus, we hypothesize:

*H2: Strong digital vision is positively related to leader-member exchange.*

Li et al. (2020) describe LMX as a key mechanism that implies feelings of trust. Further, Den Hartog et al. (2002) found that trust in the focal leader is a precondition for developing more generalized trust in management. Past research provided evidence that a high-quality LMX enhances employee trust in leaders, resulting in increased levels of trust in management (Den Hartog et al. 2002; Wong et al. 2003). Employees who have a high-quality LMX have a lower resistance to change and are better integrated into the leader’s network compared with employees who have lower-quality LMX (van Dam et al. 2008).

Thus, based on past research (Fulmer and Gelfand 2012; Mayer et al. 1995; McCauley and Kuhnert 1992; Poon 2013), we expect that LMX, in terms of an employee’s positive perceptions that management is benevolent, is required to strengthen employees’ cooperative and trusting attitudes toward management’s digitalization agenda in terms of increasing their TMD. Hence, we hypothesize:

*H3: Leader-member exchange is positively related to trust in management regarding digitalization.*

Drawing on H1 and the preceding argumentation, we expect that SDV promotes high-quality LMX, which, in turn, fosters employee TMD. Subsequently, we hypothesize:

*H4: Leader-member exchange partially mediates the relationship between strong digital vision and trust in management regarding digitalization.*

### The mediating role of POP

In the digital labor context, POP is described as an employee’s perception that management neglects moral and ethical principles, such as fairness and justice, regarding the organization’s goals, directions, and other organizational parameters regarding digitalization and the use of the technology linked to it (Boudrias et al. 2021; Colquitt et al. 2007; Fulmer and Gelfand 2012).

Thus, POP is considered an indicator of managerial malfunctioning regarding digitalization, resulting from decisions made by organizational actors based on incomplete and inaccurate information (Dean and Sharfman 1996; Eisenhardt and Bourgeois 1988). This indicator of managerial malfunctioning may negatively affect employees’ feelings about the organization (Kacmar and Carlson 1997). Further, POP is of major importance in the digital labor context because turbulent times, like the organizations’ digital transformation, could provoke cutthroat political action (Kurchner-Hawkins and Miller 2006).

Referring to the purpose of this study, we assume that the articulation of an SDV reduces employees’ POP. In particular, we expect that if management is perceived as being able to provide an SDV, employees will believe that fairness and justice principles are ensured and in line with the organization’s goals regarding digitalization (Ferris and Kacmar 1992; Hochwarter et al. 2003). An SDV based on shared values provides a direction for employees, aligns interests, and increases transparency (Choi 2007; Dean and Sharfman 1996; Eisenhardt and Bourgeois 1988; Niehoff and Moorman 1996). Hence, offering an SDV reduces employees’ POP (Bass 1985; Jamil and Naseer 2011). Consequently, the following is hypothesized:

*H5: Strong digital vision is negatively related to perceptions of organizational politics.*

In turn, employees with low POP are likely to perceive increased levels of organizational justice and support (Cropanzano et al. 1995) and also higher levels of trust (Ferris et al. 2002). The perceived inequity regarding resource allocation was shown to give rise to perceiving ongoing relationships as unfair (Bedi and Schat 2013) and to distrust management (Gotsis and Kortezi 2010). Previous results showed that political behavior was negatively related to trust, and the development of trust is inhibited in employees whose work environment was negatively affected by POP (Ferris and Kacmar 1992; Kacmar and Carlson 1997). This finding was supported by researchers who stated that POP, in general, indicates a lack of organizational trust (Bedi and Schat 2013; Davis and Gardner 2004). Although prior research focused on trust in co-workers (Ferris et al. 2002) and trust in management (Gotsis and Kortezi 2010), there is a lack of knowledge regarding TMD. Derived from prior research (e.g.,Poon 2013) we expect that low levels of integrity in terms of high levels of POP decrease employee TMD. Consequently, we hypothesize:

*H6: Perceptions of organizational politics are negatively related to trust in management regarding digitalization.*

In addition, the purpose of the current study was to investigate a lack of knowledge regarding the indirect effects of POP. Hence, based on hypothesis 1, we expect that SDV decreases employees’ POP and thereby facilitates their TMD. Thus, we posit:

*H7: Perceptions of organizational politics partially mediate the relationship between strong digital vision and trust in management regarding digitalization.*

### Double-mediation effect of LMX and POP

**Fig. 1:**
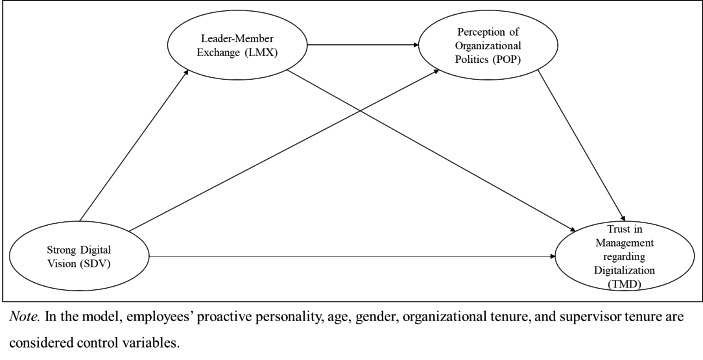
Double-mediation model of employee trust in management regarding digitalization

Building on prior research (e.g., Colquitt et al. 2007; McCauley and Kuhnert 1992; Poon 2013) and extending the theoretical framework of Mayer et al. (1995), we assume that the intercorrelation between ability in terms of SDV, benevolence in terms of LMX, and integrity in terms of POP is highly relevant to enhance employee TMD. Therefore, we suggest that the relationship between SDV and TMD is further sequentially double-mediated by LMX and POP. While SDV is assumed to promote high-quality LMX (H2), employees with high-quality LMX have lower levels of POP than employees with low-quality LMX. In this regard, previous research has shown that while management’s ability is judged by employees rather quickly and reliably, it takes more time and attention to evaluate management’s benevolence in terms of LMX as well as its integrity in terms of POP (Colquitt and Salam 2009). Moreover, employees with high-quality LMX perceive greater levels of fairness and are less affected by political tactics than employees with low-quality LMX (Bedi and Schat 2013; Ferris and Kacmar 1992; Kacmar et al. 1999; Kacmar et al. 2007; Valle and Perrewe 2000). Hence, we expect that high-quality LMX reduces employees’ POP. In turn, low levels of POP promote employee TMD (H6). As prior research has failed to investigate specific relationships between the antecedents of the model of trust (Mayer et al. 1995), we address a huge existing research gap by assuming:

*H8: Leader-member exchange and perceptions of organizational politics sequentially double-mediate the relationship between strong digital vision and trust in management regarding digitalization.* (Fig. 1)

## Method

### Participants and procedure

Online survey data were collected from 1,305 employees of an internationally operating energy supplier in Germany (response rate = 61%). Through data cleansing, we eliminated datasets of employees whose work environment was not affected by digitalization. Further datasets of employees who revealed they did not have a supervisor and those who had been working with their supervisors for less than six months were excluded. Additionally, missing values in metric scales were replaced by the mean for datasets with fewer than 5% of missing values. In the remaining 1,145 data sets (final response rate = 54%), 272 (23.8%) employees were female, 862 (75.3%) were male, nine (0.8%) were of non-binary gender, and two (0.2%) did not give this information. The average age of participants was 43 years old (*SD* = 11.97), had an average organizational tenure of 20.85 years (*SD* = 12.55) and had worked with their current supervisor for an average of 3.53 years (*SD* = 0.92).

### Measures

Following Hu and Jiang (2018) and Heggestad et al. (2019), among others, we used a back-translation procedure (Brislin 1980) for all items, except for those of LMX, to develop a German questionnaire. For LMX, we used a validated German scale. All items were measured on a five-point Likert scale ranging from 1 (strongly disagree, i.e., low) to 5 (strongly agree, i.e., high).

***TMD*** was measured by adapting a five-item scale developed by Stanley et al. (2005) to the particular context of digitalization. A sample item is “I am willing to follow management’s lead even in risky situations,” which was adapted to “I am willing to follow management’s lead even in risky digitalization situations.” The scale developed by Stanley et al. (2005) is grounded on the definition of trust developed by Mayer et al. (1995) and was found reliable and valid to assess trust in management (Stanley et al. 2005). One item (tm_3) was omitted because of low factor loadings and low correlation with the remaining items, which indicated unreliable measures. Cronbach’s alpha was 0.77.

***SDV*** was evaluated by adapting the three-item scale of “strong vision” developed by Choi (2007) to the digital labor context. Every item was modified to include “digital vision.” For example, “Our division provides a convincing vision for employees” was adapted to “Our division provides a convincing digital vision for employees.” Cronbach’s alpha was 0.76.

***LMX*** was assessed using the seven-item scale from Graen and Uhl-Bien (1995), which was translated into German by Schyns (2002). A sample item is “How well does your leader understand your job problems and needs?” Cronbach’s alpha was 0.94.

***POP*** was measured by adopting the six-item scale developed by Hochwarter et al. (2003) and querying employees’ overall perceptions concerning organizational politics. A sample item is “There is a lot of self-serving behavior going on.” Cronbach’s alpha was 0.90.

***Control variables*** in terms of employees’ proactive personalities, tenures with their supervisor and their organization, genders, and ages were considered in our model based on prior research (Gomez and Rosen 2001; Jones et al. 2010; Kim et al. 2018; van Dam et al. 2008; Velez and Neves 2018; Xiao and McCright 2015).

### Analyses

Following the approaches used in previous research (Hu and Jiang 2018; Kauppila 2016; Zhang et al. 2012), we conducted confirmatory factor analyses (CFA) in AMOS 26 to establish the construct and discriminant validity of the measurement model. To test the double-mediation model, we used bootstrap-based analysis in SPSS Process (Hayes 2013). A bootstrap sample of 5,000 was calculated to identify the significance of conditional indirect effects. Bias-corrected confidence intervals (*CI*s) and the stepwise procedure were ensured to addressing several weaknesses associated with the Sobel test (Preacher and Hayes 2008; Shrout and Bolger 2002; Zhao et al. 2010). In line with Crawford et al. (2019), we used the Huber-White sandwich estimator (White 1980) to control for structure in the data because several respondents reported to a common supervisor, implying nonindependence in the data.

## Results

The results of the CFA confirmed that the four-factor measurement model had a superior fit to all competing models (see Table 1). All items loaded significantly on their respective latent factors, supporting the construct and discriminant validity of the measures. Thus, the variables of interest to be treated as distinct constructs in the following analyses (Hu and Jiang 2018; Zhang et al. 2012).

**Table 1:**
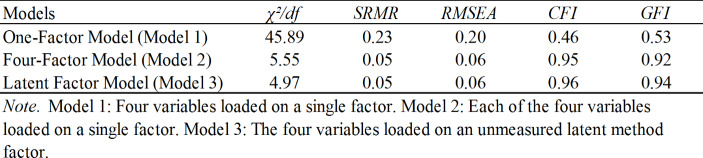
Results of the CFA

Because we collected single-source data and self-reported data from the respondents, the issue of common method variance (CMV) was addressed by using procedural and statistical remedies (Chang et al. 2010; Podsakoff et al. 2003). Procedural remedies were applied in the ex-ante research design. Specifically, we ensured the respondents’ anonymity, minimized respondents’ evaluation apprehension, balanced the order of predictor and criterion variables, and enhanced the wording of each item (Podsakoff et al. 2003). Then, Harman’s single-factor test was employed as an ex-post statistical remedy (Chang et al. 2010; Podsakoff et al. 2003). The CFA results indicated that the suspicion of CMV was minimized (see Table 1; Podsakoff et al. 2003). The results of an exploratory factor analysis showed that no single factor emerged, and one general factor failed to explain most of the variance (four factors ranging from 6.10 to 36.33% explained far less than 50% of the total variance). Next, CMV was investigated by adding an unmeasured latent method factor to our proposed four-factor model (Castanheira 2016; Hu and Jiang 2018). All items loaded on their respective theoretical constructs as well as on the latent methods factor, as proposed by Podsakoff et al. (2003). This model showed a good fit to the data (see Table 1). Castanheira (2016), the CFI difference was calculated to contrast this model to the five-factor model. The change in CFI was below the rule of thumb of 0.05, as proposed by Bagozzi and Yi (1990). Based on these findings, CMV was not a major problem in the present study.

Table 2 presents the means, standard deviations, correlations, and reliabilities of the variables of interest.

**Table 2:**
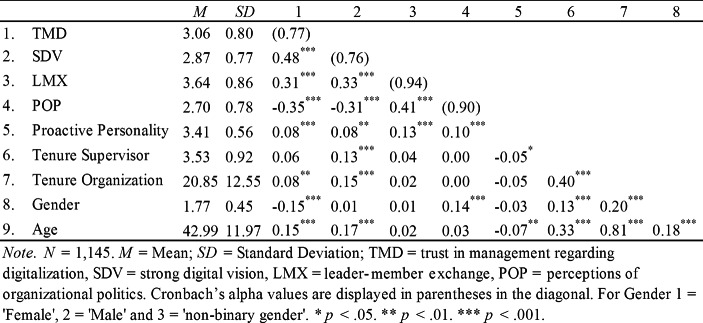
Means, standard deviations, correlations, and reliabilities of the variables of interest

The results for the double-mediation model are presented in Table 3. Regarding H1, a positive relationship between SDV and TMD was confirmed (*B* = 0.37, *p* < 0.01). Similarly, SDV was positively significantly related to LMX (*B* = 0.36, *p* < 0.01), supporting H2. Our results further provided support for H3, suggesting a positive and significant relationship between LMX and TMD (*B* = 0.09, p < 0.01). Moreover, the indirect effect of SDV on TMD through LMX (H4) was significant at 0.03 (95% bias-corrected *CI* = 0.01, 0.05). Although LMX was entered into the regression, the direct effect of SDV on TMD remained highly significant, confirming the partial mediating effect of LMX regarding the positive relationship between SDV and TMD. Thus, H4 was supported.

**Table 3:**
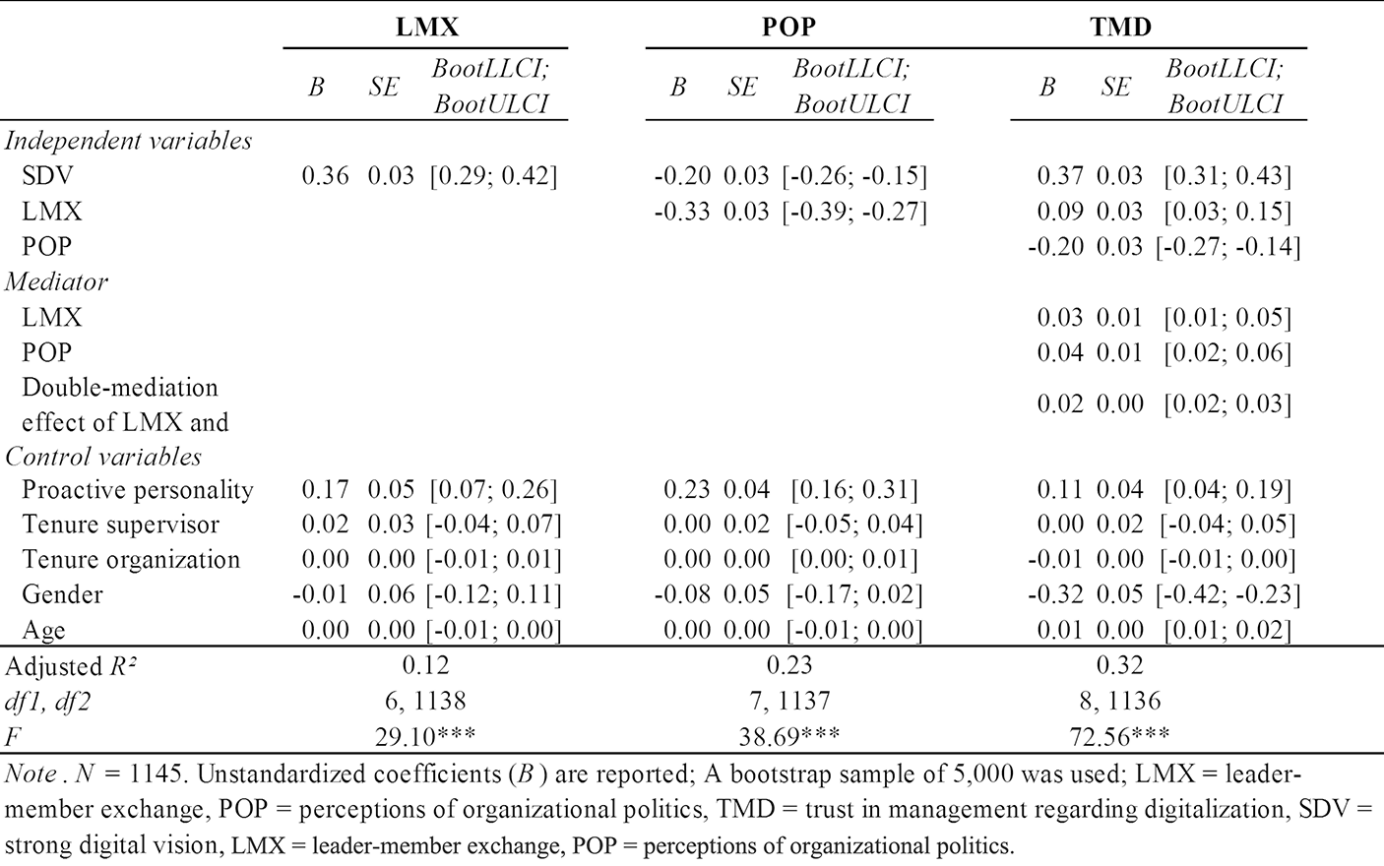
Results of the bootstrap-based regression analyses predicting LMX, POP and TMD

The results for H5 confirmed that SDV was significantly negatively related to POP (*B* = -0.20, *p* < 0.01). Moreover, H6 was supported by the negative significant relationship between POP and TMD (*B* = -0.20, *p* < 0.01). Regarding H7, the partial mediating effect of POP in the relationship between SDV and TMD was significant at 0.04 (95% bias-corrected *CI* = 0.02, 0.06). Even though POP was entered into the regression, the direct effect of SDV on TMD remained highly significant, confirming the partially mediating effect of POP in the corresponding positive relationship. Hence, H7 was supported.

Last, regarding the double-mediation model, 32% of the variance in employees’ TMD is accounted for in the independent variables in our model. The results supported that LMX and POP sequentially double-mediate the positive relationship between SDV and TMD, supporting H8. Although the double-mediation effect was significant at 0.02 (95% bias-corrected *CI* = 0.02, 0.03), the effect was weak in our model. Thus, LMX and POP had a weak significant sequential double-mediation effect on the relationship between SDV and TMD.

## Discussion

By providing empirical evidence regarding the antecedents of TMD, our study addressed research gaps of particular importance.

In line with prior studies (e.g., Uhl-Bien et al. 2000; Venus et al. 2019), we found empirical evidence that an SDV was positively related to TMD (H1). In line with previous research (Fulmer and Gelfand 2012; Kauppila 2016), SDV promoted employees’ positive perceptions about managements’ benevolence in terms of high-quality LMX, confirming H2. Furthermore, high-quality LMX promoted TMD, supporting H3. Regarding H4, we found that LMX partially mediated the relationship between SDV and TMD, although this mediation was weak (see Table 3).

Because digitalization can provoke cutthroat political actions (Kurchner-Hawkins and Miller 2006), POP was also considered in our model. We confirmed prior research by finding that a vision, in terms of SDV, reduced POP (Bass 1985; Jamil and Naseer 2011) and supported H5. Furthermore, derived from prior research (Bedi and Schat 2013; Cropanzano et al. 1995; Gotsis and Kortezi 2010), POP was associated with negative outcomes for organizations in terms of being negatively related to TMD (H6). In addition, we found empirical support that the relationship between SDV and TMD is partially mediated through POP (H7), although the partial mediation effect was weak.

Finally, regarding H8, our results provided evidence for the proposed double-mediation model showing that LMX and POP sequentially double-mediated the relationship between SDV and TMD. Our assumption was derived from research by Mayer et al. (1995), among others, and has been extended and applied to the digital labor context in the current study. In particular, managements’ ability to articulate a clear digital vision in terms of SDV promotes the perception that management is benevolent, which implies high-quality LMX between leaders and followers. Employees’ perceptions about management’s support for any loyalty regarding digitalization promotes employees’ perceptions that management ensures fairness and justice regarding digitalization in terms of reducing employees’ POP. Consequently, reduced levels of POP result in higher levels of TMD. Although we found a significant double-mediating effect of SDV on TMD through LMX and POP, this effect was weak. This poor overall indirect effect might be due to conflicting indirect effects of LMX and POP that may cancel each other out or it might depend on the variance in the sample (Agler and Boeck 2017). Future research should investigate whether the double-mediation effect of LMX and POP on the relationship between SDV and TMD becomes stronger as the variance of the sample increases.

It should be noted that all direct relationships in the model were strong and significant. Thus, referring to the purpose of this study, crucial antecedents of employee TMD in the digital era have been identified, and existing research, like the model of trust by Mayer et al. (1995), has successfully been specified, extended and applied to the context of digitalization.

### Managerial implications

Our empirical findings call attention to the importance of employee TMD in the digital labor context (Kotter 1995; van Dam et al. 2008). Organizations might rethink their existing institutional aspects and strategic approach to the implementation of digitalization. Therefore, our results can guide organizations in the development of TMD. In this regard, SDV in terms of ability, LMX in terms of benevolence, and POP in terms of integrity, were unique and significant antecedents of TMD and thus provide cornerstones for the creation of employee TMD. Furthermore, our research provides new empirical evidence that these antecedents should be considered as a chain of factors which are in a specific relationship to each other. While the model proposed by Mayer et al. (1995) provided a fundamental theoretical framework for our own model, we addressed the need to look more closely at the mediation effects regarding the development of TMD.

To directly alter employee TMD, management should provide an SDV for employees. SDV can serve as an institutionalized socialization tactic to reduce employees’ digitalization anxiety by offering information, guiding behavior, aligning interests, and creating a sense of community in the new digital environment (Kim et al. 2005; Reid and Roberts 2011), thus encouraging TMD. So, we suggest developing and increasing management’s abilities related to SDV, for example by continuously critically questioning the existing abilities and capabilities of management in the digital context (Shah et al. 2017). In this regard, training strategies for management are helpful to build specific expertise regarding the formulation and articulation of an SDV (Arthur et al. 2003; Burnett et al. 2009; Colquitt et al. 2007). More precisely, managements’ ability to share a common vision about the organizational future is the main challenge for leadership in these times, since both management and employees are continuously confronted with the need to adapt to change regarding ongoing digitalization (Cortellazzo et al. 2019; Horner-Long and Schoenberg 2002).

Another important aspect is the recruitment of suitable managers as well as leaders and employees (Cortellazzo et al. 2019). Thereby, organizations should hire strategically by paying particular attention to the alignment of individuals with the current SDV (Shah et al. 2017).

Considering benevolence in terms of LMX and integrity in terms of POP, team-building programs with a particular focus on the digital labor context may benefit the relationship between employees and their leaders as well as employees’ POP. Further, relationships between employees and leaders are strengthened through the articulation of managements’ digital vision in terms of an SDV (Cortellazzo et al. 2019).

Additionally, through particular training courses, managers promote their interpersonal skills (e.g., to provide feedback and open communication) to build high-quality relationships with their employees and be sensitized to POP (Colquitt et al. 2007; Shah et al. 2017). This is also of importance because management must pay attention to ongoing organizational politics, prevent the occurrence of high POP, and apply targeted interventions to reduce POP (Crawford et al. 2019).

Management has to take a holistic approach regarding organizational digitalization by further considering the intercorrelation of managements’ characteristics in terms of SDV, LMX, and POP to develop TMD. Thereby, organizations should note that an SDV shapes a collective identity among employees that facilitates high-quality LMX (Kauppila 2016). Particularly, an SDV will promote employees’ perceptions regarding management’s supportiveness, loyalty, and openness regarding digitalization. As a consequence, high-quality LMX relationships imply desired dyadic relationships between employees and leaders (Kauppila 2016) that increase employees’ perceptions regarding compliance to fairness and justice principles within organizations in terms of low POP. Subsequently, employee TMD is promoted.

Thus, in the digital labor context, management increases employee TMD by specifying an SDV that addresses the importance and implementation of high-quality LMX and fair policies within the organization and limiting the degree to which decisions are politically driven (Crawford et al. 2019). Management should use their SDV to create transparency and promote open communication with employees regarding decision-making in digitalization agendas to increase LMX and avoid employees’ POP and thus increase their TMD.

### Limitations and future research

This study has several limitations that should be considered in future research. First, this study was not based entirely on reliable and valid scales. For example, TMD was analyzed by adapting items of the scale developed by Stanley et al. (2005) to the digital labor context. Thus, further research is needed to validate our measures.

Second, all measures were focused on employees’ perceptions to emphasize their role in the corporate challenge of digitalization. This focus was based on the theoretical assumptions of Mayer et al. (1995) as well as on previous research that found, for example, regarding LMX, that the emergence of a good mutual relationship is dependent on employees (Graen 2003; Graen and Scandura 1987; Schyns 2016).

Because we used self-reporting measures and single-source data, our study design partly accounted for CMV bias (Chang et al. 2010; Podsakoff et al. 2003). By successfully performing procedural and statistical remedies, the risk of CMV was reduced to an extent. Nevertheless, we recommend that in future research different perceptions and querying multiple raters, such as leaders, should be considered to avoid CMV.

Moreover, by applying a cross-sectional study design, the data were collected at one point in time. Due to this, we were not able to conclude causal relations based on the data (e.g., Antonakis 2017).

In line with Podsakoff et al. (2003), we recommend future research to verify the hypothesized relationships by employing a longitudinal or experimental study design.

Next, our data were collected from employees in one organization in Germany, an internationally operating energy supplier. Considering that digitalization and innovation highly affect the energy industry worldwide in terms of the energy revolution, climate change, and the related transition from conventional to renewable energies (Midttun and Piccini 2017; Zhang et al. 2017), our analyses provided new insights into a highly relevant issue in the global economy. Nevertheless, it might be of further interest to investigate the varying effects of, for example, providing a vision in various organizations and measuring the resulting effects.

The problem of endogeneity was addressed by including several control variables in our model, even though it could not be avoided entirely in the present study. Nevertheless, further variables might be worth considering to identify conditional, indirect effects that explain TMD. Following prior research, future studies could investigate perceived risk, trust regarding coworkers or organizational factors, such as leadership, organizational support, the way organizations implement digitalization, and attitudes toward digitalization (Adler and Borys 1996; Baer et al. 2018; Dirks and Ferrin 2002; Mayer et al. 1995).

## Conclusion

The present study advances the research on trust in the workplace by investigating the antecedents of TMD. In considering employee TMD as a necessary precondition for the successful implementation of digitalization and investigating institutional aspects as antecedents of TMD, our study addressed research gaps of particular importance. Based on prior research (Mayer et al. 1995; McCauley and Kuhnert 1992), we derived our research model and found empirical evidence for our hypothesized double-mediation model. We confirmed that SDV, LMX, and POP were crucial factors that promoted TMD. By extending the model of trust in terms of investigating the mediating effects, the results showed that LMX partially mediated the relationship between SDV and TMD. Specifically, providing an SDV fostered interpersonal relations between employees and leaders in terms of LMX, which in turn increased TMD. Besides, POP partially mediated the relationship between SDV and TMD. Providing an SDV in times of digitalization reduced employees’ POP, which further increased their TMD. Lastly, our study confirmed that LMX and POP sequentially double-mediate the relationship between SDV and TMD.

Consequently, to create and increase employee TMD and successfully implement digitalization within organizations, management should provide SDV, ensure high-quality LMX, and reduce POP.

The results of the present study contribute to theory development concerning TMD by specifying, extending and applying the model of trust by Mayer et al. (1995) in terms of the crucial factors of trust to the digital labor context. This study’s results provide recommendations for management in terms of identifying institutional aspects that enhance employee TMD in the digital era.
